# Cholera prevention and control in refugee settings: Successes and continued challenges

**DOI:** 10.1371/journal.pntd.0007347

**Published:** 2019-06-20

**Authors:** Kerry Shannon, Marisa Hast, Andrew S. Azman, Dominique Legros, Heather McKay, Justin Lessler

**Affiliations:** 1 Johns Hopkins Medicine, Department of Emergency Medicine, Baltimore, Maryland, United States of America; 2 Johns Hopkins Bloomberg School of Public Health, Department of Epidemiology, Baltimore, Maryland, United States of America; 3 World Health Organization, Geneva, Switzerland; Institute for Disease Modeling, UNITED STATES

## Introduction

Cholera has long been viewed as a serious threat for refugee populations [1, 2]. In the 1980s and 90s, refugee camps proliferated in Africa and Asia as a result of large civil wars and environmental disasters. These camps experienced large-scale cholera outbreaks with regularity because of overcrowding, scarce clean water, and poor sanitation and hygiene practices [2–4]. Death rates were often high because of preexisting malnutrition, comorbidities, and limited access to medical care. With appropriate clinical management, cholera mortality can be well below 1%, but it can be as high as 50%–60% without proper care [3, 5–7]. During this time, humanitarian organizations developed a variety of guidelines and standards to reduce morbidity and mortality during cholera outbreaks in these populations [8, 9]. Mobilization around these issues was greatly accelerated in 1994, when a particularly massive outbreak occurred among Rwandan refugees in the Lake Kivu region of Zaire (now the Democratic Republic of the Congo), and approximately 42,000 people died [10]. In response to this unprecedented tragedy, the humanitarian community developed and adopted the Sphere standards for the minimum acceptable living conditions and availability of health services in refugee camps and other humanitarian responses [11].

Since this time, the Sphere standards have been updated, and additional coordinating systems have been developed, including the cluster approach to humanitarian response, the Transformative Agenda, and the adaptation of United Nations High Commissioner for Refugees’ (UNHCR) refugee coordination in the context of the Transformative Agenda [12–14]. With the recent goal to reduce cholera deaths 90% by 2030 set by the Global Task Force on Cholera Control, there is a renewed urgency to examine successes and address remaining gaps in cholera control [15]. Although refugee camps continue to experience many vulnerabilities, the increased focus on improved camp coordination, preparedness, timely multisectoral response, and adherence to minimum standards has resulted in a notable decrease in the number and size of camp-based cholera outbreaks and associated mortality.

## Historical trends in cholera outbreaks in refugee camps

To illustrate these changing trends, we highlight several historic outbreaks in refugee camps as described in the scientific literature. Although it is difficult to directly compare previous refugee populations to modern ones because of sparse data and differences in refugee demography, these illustrative outbreaks provide context for the high cholera morbidity and mortality in camps in these times. We conducted a targeted review of literature published prior to 1994 containing combinations of the keywords “cholera”, “acute diarrhea”, “refugee”, “camp”, and “displaced.” Searches were done on the electronic platforms Google Scholar and PubMed, and approximately 120 articles were reviewed for relevance. There were no formal exclusion criteria. We identified eight outbreaks in refugee camps that occurred between 1971 and 1991 across Africa and Asia (Table 1). Where reported, the proportion of the refugee population affected (attack rate) was 1.8% or greater (4 outbreaks), and the proportion of cases who died (case fatality ratio [CFR]) was as high as 30% (range 1%–30%, 8 outbreaks) [16–24].

**Table 1:** Sample of cholera outbreaks in refugee camps up to 1994 in published literature.

Country	Dates	Camp Names	Country of Origin	Cases (Attack Rate)	Deaths (CFR)	Source
India	1971, May	Bongaon and others	East Pakistan (Bangladesh)	* (*)	* (30%)	Mahalanabis, 1973
Thailand	1980, Mar-May	Nong Samet, Nong Chan	Kampuchea (Cambodia)	335 (*)	6 (1.8%)	Holck, 1983
Sudan	1985 May-Jun	Shagarab East 1 and 2	Ethiopia	1,175 (4.3%)	54 (4.6%)	Mulholland, 1985
Sudan	1985 May-Nov	Wad Sherife	Ethiopia	1,793 (1.8%)	32 (1.7%)	Sorenson, 1988
Somalia	1985	Gannet and others	Ethiopia	6,560 (*)	1,069 (3%–25%)	CDC, 1992
Malawi	1988 Mar-May	Mankhokwe	Mozambique	951 (2.6%)	31 (3.3%)	Moren, 1991; CDC, 1992
Malawi	1990 Aug-Dec	Nyamithutu	Mozambique	1,931 (2.4%)	68 (3.5%)	Swerdlow, 1997; CDC, 1992
Turkey	1991 Apr-May	Cukurca, Isikveren, Uzumlu, Yekmal	Iraq	* (*)	4,958^1^ (1.2%)	CDC, 1991

*Information unknown or unavailable.

^1^Value is a maximum: 74% of 6,700 total deaths attributed to “diarrhea, dehydration, or malnutrition.”

Abbreviations: CDC, Centers for Disease Control and Prevention; CFR, case fatality ratio

Accounts of these outbreaks consistently describe inadequate water chlorination, a shortage of sanitation facilities, and improper disposal of cholera waste. In most accounts, there were considerable barriers to a timely response, and implementation of cholera control measures was significantly delayed, sometimes for weeks after the outbreak had started. In addition, a lack of cholera-specific training and supplies in health centers was cited as a contributing factor to high CFRs. Some camps fared better than others; a 1988 review found that camps that were planned and/or had an established primary healthcare system saw a more rapid reduction in mortality as compared with unplanned border camps or camps with a consistent influx of new arrivals [25].

In 1994, the largest refugee camp–related outbreak ever reported occurred following the influx of 500,000–800,000 Rwandan refugees into the cholera-endemic North Kivu Province of the Democratic Republic of the Congo [10]. Within a month, 58,000–80,000 cases of cholera occurred in the city of Goma and surrounding refugee camps, with incidence peaking at over 6,000 cases per day. The crude mortality rate was one of the highest ever recorded, at 25–35 deaths per 10,000 people per day [10], vastly exceeding the commonly accepted emergency threshold of one death per 10,000 per day [26, 27]. Numerous factors were identified as contributing to the severity of this outbreak. Because of the rapid relocation of such a large population, sufficient facilities for water and sanitation were not established in advance of refugee arrival. There was only an average of 0.2 liters of clean water per person per day during the first week of the outbreak, and rocky ground limited the ability to dig latrines [28]. These factors in addition to the general poor health of the incoming refugees and endemicity of cholera in North Kivu created ideal conditions for the spread of cholera. Furthermore, overcrowding of medical facilities and lack of training in cholera case management contributed to CFRs that approached 50% in some clinics, and an estimated 24,000 deaths occurred among people who never received care [10, 29].

Based largely on the scale of the humanitarian emergency in this region and the magnitude of mortality seen in Goma, the international community mobilized to develop strong evidence-based standards for refugee health and assistance. In 1997, the Sphere project, a collaboration between 228 humanitarian organizations from 60 countries, developed *The Humanitarian Charter*, delineating the rights, roles, and duties that should govern the response to humanitarian crises and outlining the core and minimum standards needed to achieve these goals [11]. The Sphere handbook was released in 2000 and was revised in 2004, 2011, and 2018. Though there were previous efforts to set and standardize guidelines for humanitarian response going back to the 1980s [8, 9, 25, 30, 31], the Sphere project helped to set globally applicable evidence-based minimum standards for humanitarian response that were largely accepted as feasible and achievable by the international community [32]. These standards have been widely adopted and endorsed [1, 33–35].

The most pertinent Sphere standards for cholera prevention and control are those for water, sanitation, and hygiene (WASH) (Table 2), and for health service delivery. Specific protocols were outlined for cholera preparedness, including pretraining health center staff, stocking sufficient oral rehydration solution and intravenous fluids in advance, improving facilities for sanitation and hygiene, and designating a separate area for cholera treatment centers that minimizes opportunities for transmission [34, 35]. These protocols strongly emphasize the need for surveillance to facilitate prompt diagnosis and rapid medical response. They now play an important role in the design and implementation of refugee camps administered by UNHCR, are incorporated in UNHCR epidemic preparedness and response guidance, and are included in the manuals of numerous other organizations in the humanitarian community, including Médecins Sans Frontières and the International Federation of Red Cross and Red Crescent Societies [36, 37].

**Table 2:** Key minimum Sphere standards for water, sanitation, and hygiene.

Analysis Standards	Key Indicators
Water Supply Standards	Access and water quantity • 15 liters of water per person per day • 1 water point per 250 people • Maximum distance of 500 meters to water point • Maximum of 30 minutes wait time at water source
	Water quality • ≥0.2–0.5 mg/liter of free residual chlorine at point of water delivery (chlorinated water) • <10 thermotolerant fecal coliforms per 100 ml at point of water delivery (unchlorinated water)
Hygiene Promotion Standards	Identification, access to, and use of hygiene items • 250 grams of soap per person per month for bathing • Two 10–20 liter water containers per household
Excreta Management	Access to and numbers of toilets • Maximum 20 people per toilet • Toilets no more than 50 meters from dwellings • Toilets reported as safe by women and girls
Analysis Standards	Initial assessment • Monitoring and evaluation

Since the adoption of the Sphere standards, additional changes have been made to restructure and improve humanitarian response in ways that further improve cholera outcomes in refugee camps. The Inter-Agency Standing Committee (IASC), a global humanitarian forum, adopted the “cluster approach” in 2005, which designated a global lead for nine sectors of humanitarian response including water and sanitation. The intention of these reforms was to promote partnerships between international humanitarian organizations and to strengthen the capacity of the global humanitarian response in order to increase preparedness, coordination, and accountability in emergencies [12, 38]. In 2011, the IASC implemented the Transformative Agenda, which took additional steps to improve cluster and sector management, accountability, leadership, and coordination in humanitarian response [13]. These changes have led to an organizational infrastructure better prepared and capable of managing cholera outbreaks in refugee settings. Community health programs have been developed using community health workers and hygiene promoters, and case management has improved following the designation of isolated cholera treatment centers and prepositioning of medications and supplies. In some settings, these strategies have been augmented by the use of oral cholera vaccines (OCVs).

Surveillance for cholera in refugee camps has also improved. In coordination with host government ministries of health (MOHs), UNHCR is charged with ensuring that camps are designed and managed to handle cholera outbreaks, including developing appropriate surveillance systems. To this end, suspected cholera cases in refugee camps are immediately reported to UNHCR’s internal health information surveillance system, and appropriate investigation, lab confirmation, and response activities are conducted in collaboration with local MOHs to ensure outbreaks are carefully tracked. This surveillance system, in conjunction with improvements in field and in-country lab capacity, has facilitated both early warning systems and outbreak monitoring.

## Recent trends in refugee camp cholera outbreaks

This improved surveillance allows for an in-depth examination of cholera in refugee settings. To examine current trends in cholera in refugee camps, we reviewed all probable and confirmed cholera cases and deaths reported to UNHCR from 2009 to 2016. These data were provided directly from UNHCR’s health information surveillance system under an ongoing data sharing agreement. For this review, data were included from registered refugee camps, settlements, and sites. Urban settings where refugees were integrated into the community were excluded.

For each outbreak, the attack rate and CFR were calculated when possible. The population in each camp was derived from UNHCR outbreak reports if available and from the World Health Organization (WHO) and nongovernmental organizations if not reported by UNHCR (see Table in S1 Table). For cases in which camp population was reported to change over the course of the outbreak, a weighted average of the population was calculated for all available weeks of the outbreak, excluding weeks with zero cases at the beginning or end of an outbreak. If no specific weekly/monthly data were available, yearly population data for camp size were used from the UNHCR population statistics database (http://popstats.unhcr.org/en/demographics). Cases were aggregated by camp and plotted on a map using ArcGIS version 10.2 (ESRI, Redlands, CA, United States of America) and open-source Global Administrative Areas (GADM) shape files to delineate country boundaries [39].

**Fig 1:**
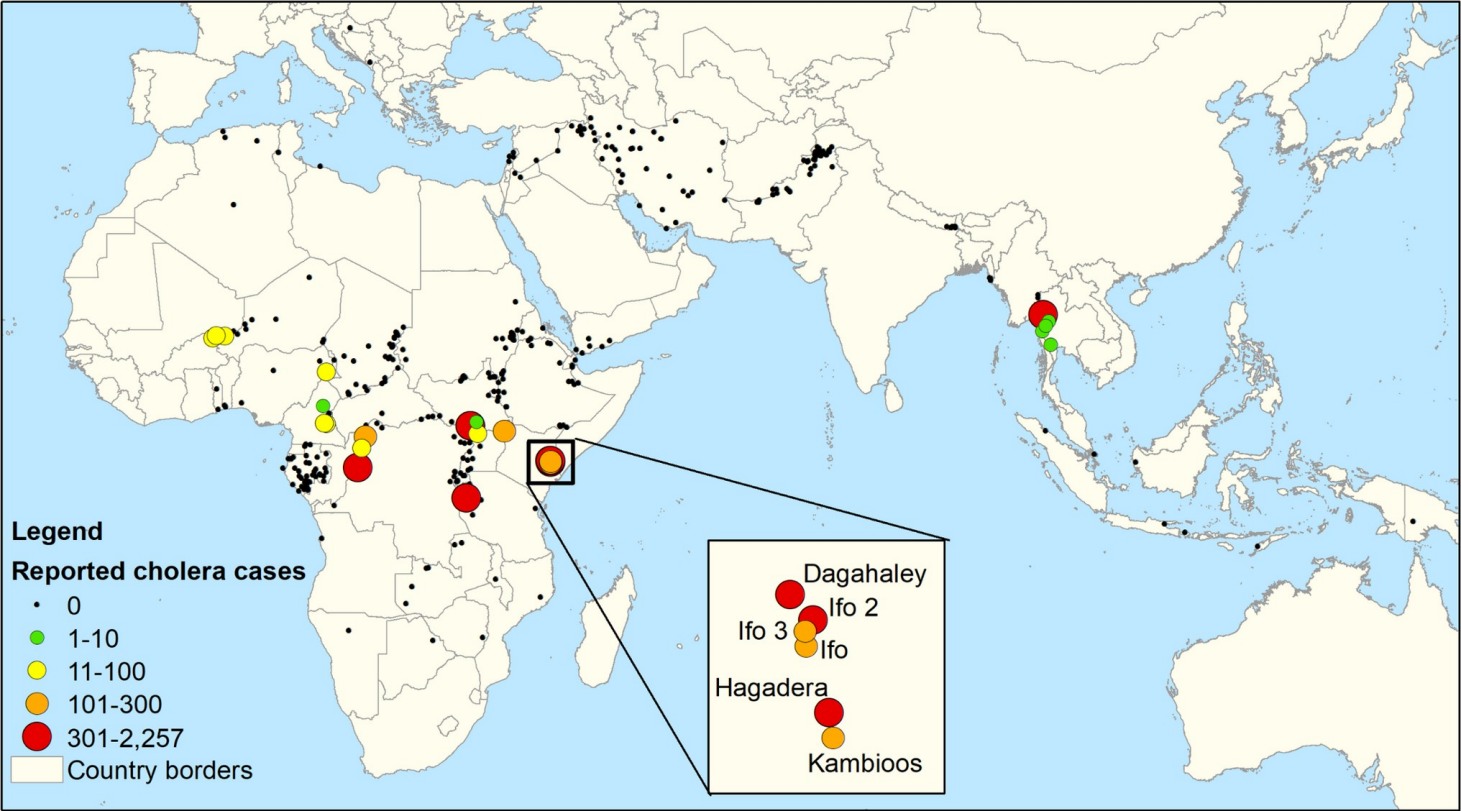
Map of reporting UNHCR locations and cumulative reported cholera count, 2009–2016 (created in ArcGIS version 10.2 using GADM shape files found at gadm.org). GADM, Global Administrative Areas; UNHCR, United Nations High Commissioner for Refugees.

Of the approximately 500 refugee camps and sites required to report to UNHCR, 26 reported confirmed cholera cases between 2009 and 2016. These 26 camps individually reported 38 outbreaks, totaling 8,034 confirmed and suspected cases (range: 1–2,257 cases per outbreak) and 69 deaths (range: 0–14 deaths per outbreak), with an overall CFR of 0.9% (Table 3). Across all outbreaks, CFRs did not exceed 2% in outbreaks with more than 75 cases, and the majority of outbreaks had no mortality due to cholera. For instance, Ifo camps 1, 2, and 3 in Kenya had a combined 485 cases in 2011, and Mae La camp in Thailand had 633 cases in 2010, both with no reported mortality. The median attack rate across outbreaks was 21.4 cases per 10,000 (range: 0.9–451.4 per 10,000). Thirty-three out of the 38 outbreaks occurred in Africa, and 18 were in Kenya alone, largely among subcamps in the Dadaab camp complex (Fig 1). Thailand was the only country outside of Africa that reported cholera in refugee camps. Tanzania had the most documented cases, at 3,620, 97% of which were reported from three camps in 2015 in conjunction with a massive countrywide epidemic [40]. Kenya had the second-highest number of cases (2,908), followed by Thailand (645) and the Republic of the Congo (628).

**Table 3:** Cholera outbreaks in refugee camps (2009–2016).

Country	Refugee Camp	Start Date of Outbreak	End Date of Outbreak	Cases	Deaths	Case fatality ratio (%)	Estimated Population	Attack Rate (Cases per 10,000)
Cameroon	Bazzama	10/6/2011	10/13/2011	13	1	7.7	785	165.61
Cameroon	Bertoua	10/6/2011	10/13/2011	13	1	7.7	-	-
Cameroon	Gado	11/2/2014	11/9/2014	4	0	0.0	17,594	2.27
Cameroon	Minawao	8/3/2014	10/12/2014	54	5	9.3	24,667	21.89
Kenya	Dagahaley	9/20/2011	1/5/2012	263	3	1.1	126,214	20.84
Kenya	Dagahaley	8/1/2015	5/8/2016	644	3	0.5	87,131	73.91
Kenya	Hagadera	8/25/2011	1/26/2012	170	3	1.8	139,805	12.16
Kenya	Hagadera	9/14/2012	10/26/2012	22	0	0.0	139,415	1.58
Kenya	Hagadera	8/8/2015	6/3/2016	814	6	0.7	105,950	76.83
Kenya	Ifo	9/4/2011	1/26/2012	175	0	0.0	124,832	14.02
Kenya	Ifo	9/25/2012	10/30/2012	11	0	0.0	75,356	1.46
Kenya	Ifo	5/19/2013	5/26/2013	9	0	0.0	100,056	0.90
Kenya	Ifo	8/23/2015	7/11/2016	56	0	0.0	83,950	6.67
Kenya	Ifo 2	9/4/2011	1/20/2012	200	0	0.0	65,442	30.56
Kenya	Ifo 2	4/21/2013	5/26/2013	16	0	0.0	64,789	2.47
Kenya	Ifo 2	8/7/2015	4/25/2016	93	1	1.1	49,940	18.62
Kenya	Ifo 3	9/11/2011	1/17/2012	109	0	0.0	37,115	29.37
Kenya	Kakuma	9/14/2009	11/30/2009	162	3	1.9	62,015	26.12
Kenya	Kambioos	10/27/2011	2/10/2012	56	0	0.0	11,361	49.29
Kenya	Kambioos	7/16/2015	4/18/2016	96	1	1.0	19,671	48.80
Niger	Ayorou	7/1/2012	7/15/2012	11	0	0.0	9,189	11.97
Niger	Mangaize	7/31/2012	9/18/2012	14	0	0.0	5,549	25.23
Niger	Mangaize	5/13/2013	5/13/2013	1	1	100.0	8,004	1.25
Niger	Tabareybarey	5/7/2013	5/24/2013	29	0	0.0	8,819	32.88
Republic of Congo	Betou	1/25/2012	6/6/2012	256	6	2.3	37,333	68.57
Republic of Congo	Impfondo	1/27/2012	6/1/2012	61	9	14.8	21,140	28.86
Republic of Congo	Liranga	1/2/2012	10/1/2012	311	6	1.9	19,396	160.34
South Sudan	Gorom	6/26/2015	7/1/2015	5	0	0.0	2,754	18.16
Tanzania	Nyarugusu	9/30/2009	11/9/2009	116	1	0.9	60,971	19.03
Tanzania	Nyarugusu	5/14/2015	6/3/2015	566	1	0.2	155,000	36.52
Tanzania	Kagunga	5/10/2015	5/27/2015	2,257	14	0.6	50,000	451.40
Tanzania	Kigoma transit center	5/19/2015	5/27/2015	681	3	0.4	-	-
Thailand	Don Yang	10/6/2011	10/6/2011	3	0	0.0	4,144	7.24
Thailand	Mae La	5/24/2010	11/8/2010	633	0	0.0	46,078	137.38
Thailand	Nu Po	9/20/2015	10/11/2016	3	0	0.0	10,461	2.87
Thailand	Tham Hin	10/16/2011	10/16/2011	2	1	50.0	7,796	2.57
Thailand	Umpiem Mai	4/26/2010	4/26/2010	7	0	0.0	17,697	3.96
Uganda	Adjumani	8/9/2016	9/25/2016	98	0	0.0	30,000	32.67
**Overall**	-	**9/14/2009**	**10/11/2016**	**8,034**	**69**	**0.9**	**1,559,243**	**47.07**

## Discussion

The dramatic reduction in the size of cholera outbreaks in refugee camps and associated mortality indicates the notable impact of the more systematic approaches to cholera preparedness and control adopted in recent decades. No single camp outbreak had more than 14 deaths, and the overall CFR across outbreaks was below the 1% standard for appropriate clinical management. Cholera-associated mortality in many of these camp settings was lower than outbreaks in nonhumanitarian settings from the same period, including within the same countries [41–44]. The CFR in refugee camps across outbreaks was also lower than the overall cholera CFR from the African or Southeast Asian regions at this time [45]. Furthermore, attack rates were below 1% in all but two outbreaks, as compared with attack rates of 2%–10% among camp outbreaks with known population data prior to 1994. These low attack rates are also noteworthy because many of the modern refugee populations originated from or settled in cholera-endemic areas.

These results highlight both the successes and continued importance of enhanced cholera preparedness, response, and multisectoral coordination. Refugee camps remain vulnerable to cholera introductions in part because of the health risks associated with displacement, overcrowding, and inadequate initial water and sanitation conditions among incoming populations. However, as these data indicate, timely and appropriate camp planning, preparedness, coordination with local MOHs, and adherence to minimum WASH standards can greatly reduce the propensity for cholera to spread. Appropriate training and case management can furthermore greatly reduce mortality in the setting of an outbreak, in contrast to prior epidemics in which CFRs were sizable. Adherence to WASH and health standards have furthermore been directly associated with decreased overall mortality and mortality under five years old in refugee camps, particularly in the context of diarrheal disease [33, 46]. In light of the upcoming 2030 goals, these lessons are vitally important for ongoing and expanding policies for cholera preparedness and control.

Despite these overall improvements, significant challenges remain for cholera prevention and control in refugee camps. Although conditions have greatly improved in most refugee settings, there have been situations in which the severity of a crisis has impeded preparedness or timely adherence to minimum standards. In these instances, if cholera is introduced to the camp, there is increased vulnerability to a larger outbreak. For example, a large epidemic of 973 cases occurred following the 2011 famine event in eastern Africa, when more than 200,000 Somalian refugees arrived in the Dadaab camp complex in Kenya [47–49]. This outbreak was characterized by large influxes of new arrivals, severe malnutrition, flooding, challenges to clean water access, and security concerns, all of which overwhelmed response efforts [50]. Another example occurred in camps in Tanzania in 2015, when a large countrywide cholera outbreak of nearly 22,000 cases overwhelmed general response efforts and caused 3,504 cases among refugees [51]. In this case, cholera likely did not originate in the refugee population but was associated with the general epidemic in the host country [52, 53]. Of note, the CFR among refugees in Tanzania was 0.5% compared with 1.6% countrywide, indicating the potentially higher degree of cholera preparedness and control within the camps compared with the country as a whole.

A similar population is camps of internally displaced persons (IDPs), who are displaced within the borders of their country of origin. Because of different regulations regarding these populations, UNHCR does not have a general or exclusive mandate over IDPs, and local governments do not always allow humanitarian organizations unrestricted access to these populations [54]. As a result, cholera preparedness and response are not always standardized in IDP camps to the same extent as in refugee camps, potentially leaving them more vulnerable to cholera introductions and outbreaks. Consequently, crude mortality rates in IDP camps have remained higher than refugee camps on average, and large and severe cholera outbreaks continue to occur [26]. Recent noteworthy examples include Yemen, which recorded over 1 million cholera cases among IDPs and other citizens from 2016 to 2018 [55, 56], and South Sudan, which reported more than 6,000 cases in 2014 [57]. The humanitarian community will need to overcome greater political and logistical challenges when serving these communities in order to meet the 2030 goals; however, the successes of cholera control in refugee camps indicate that similar efforts to implement WASH and health standards have the potential to greatly benefit IDP populations and improve cholera outcomes.

For these situations in which crises may overwhelm the capacity to provide appropriate services and the risk of cholera is thought to be high, the use of the OCV is an emerging strategy, endorsed by WHO, to supplement other cholera prevention and control efforts [58]. In 2013, WHO established a global stockpile of OCV, which was first used in an emergency setting in 2014 in two IDP camps in South Sudan [57]. As of July 2017, more than 25 million doses have been deployed from this stockpile, and availability has been increasing each year [59]. Although investment in water, sanitation, and healthcare infrastructure, supplies, and service provision will have the most impact on prevention and control of cholera and other diarrheal diseases, the vaccine is a potentially powerful tool that can be used when minimum standards are challenging to meet in a timely manner because of the severity or complexity of a humanitarian emergency.

There were several limitations to this analysis. Determining accurate population sizes, case counts, and attack rates is challenging in refugee settings, and there were several potential sources of error. Utilizing different sources of population data may introduce biases due to potential variability in data collection methods, which could lead to over- or underestimation of camp populations. Reporting to UNHCR might also have been imperfect, leading to over- or underestimation of cases, particularly in small outbreaks in which cholera may never have been identified. In larger outbreaks, cases might have been double counted because of high mobility of refugees in emergency situations, and cases might have been missed because of overcrowding, lack of presentation to healthcare facilities, or failure to report cases to UNHCR. For these reasons, reported case counts may be higher or lower than reported in other sources.

In addition, only refugees in registered camps, settlements, or sites were included in this analysis. This excludes refugees in urban settings and those integrated into local communities, which is a growing proportion of the refugee population. Furthermore, it is problematic to directly compare modern refugee populations to those in the 1970s and 80s because of drastic changes in disease reporting systems and the changing demographics of refugee populations. For instance, outbreaks represented in the literature from this time might be larger or more severe than the average. Despite these challenges, the available evidence strongly indicates the trends toward decreased cholera transmission and mortality in refugee camps as noted in this paper.

## Conclusions

Cholera continues to be a significant problem in humanitarian settings, with recent outbreaks in displaced populations in South Sudan, Yemen, Cameroon, Nigeria, Tanzania, Uganda, Haiti, and Iraq. The success of cholera prevention and control in refugee camps over the past 2 decades highlights the possibility of managing this deadly disease, even in complicated humanitarian crises. Sphere standards and associated control strategies have been shown to be effective in humanitarian crisis settings, dramatically reducing the number and size of outbreaks seen in refugee camps after the North Kivu disaster. Although other vulnerable populations, particularly IDPs, continue to suffer from a substantial cholera burden, application of these strategies in combination with supplementary tools such as OCV have the potential to substantially reduce cholera cases and deaths in line with the 2030 goal of reducing cholera deaths by 90% worldwide.

## Supporting information

S1 Table Sources of refugee camp population data used to calculate attack rates. (DOCX)
