# Supplementary material for: Cholera prevention and control in refugee settings: Successes and continued challenges
Source: PLoS Negl Trop Dis. 2019 Jun 20;13(6):e0007347. doi: 10.1371/journal.pntd.0007347 (PMC6586254; doi:10.1371/journal.pntd.0007347)
Supplement: S1 Table — (DOCX) [file pntd.0007347.s001.docx]

Table S1: Sources of refugee camp population data used to calculate attack rates

| **Country** | **Refugee Camp** | **Start date of Outbreak** | **End date of Outbreak** | **Estimated Pop. Size** | **Reference for population estimate** |
| --- | --- | --- | --- | --- | --- |
| Cameroon | Bazzama | 10/6/2011 | 10/13/2011 | 785 | http://popstats.unhcr.org/en/demographics |
| Cameroon | Bertoua | 10/6/2011 | 10/13/2011 | - |  |
| Cameroon | Gado | 11/2/2014 | 11/9/2014 | 17,594 | UNHCR outbreak report |
| Cameroon | Minawao | 8/3/2014 | 10/12/2014 | 24,667 | [UNHCR outbreak report](http://www.unhcr.org/54536b9b9.html) |
| Kenya | Dagahaley | 9/20/2011 | 1/5/2012 | 126,214 | <http://data.unhcr.org/horn-of-africa/region.php?id=3&country=110> |
| Kenya | Dagahaley | 8/1/2015 | 5/8/2016 | 87,131 | <http://data.unhcr.org/horn-of-africa/region.php?id=3&country=110> |
| Kenya | Hagadera | 8/25/2011 | 1/26/2012 | 139,805 | <http://data.unhcr.org/horn-of-africa/region.php?id=3&country=110> |
| Kenya | Hagadera | 9/14/2012 | 10/26/2012 | 139,415 | [UNHCR report](http://data.unhcr.org/horn-of-africa/region.php?id=3&country=110) |
| Kenya | Hagadera | 8/8/2015 | 6/3/2016 | 105,950 | <http://data.unhcr.org/horn-of-africa/region.php?id=3&country=110> |
| Kenya | Ifo | 9/4/2011 | 1/26/2012 | 124,832 | <http://data.unhcr.org/horn-of-africa/region.php?id=3&country=110> |
| Kenya | Ifo | 9/25/2012 | 10/30/2012 | 75,356 | [UNHCR report](http://data.unhcr.org/horn-of-africa/region.php?id=3&country=110) |
| Kenya | Ifo | 5/19/2013 | 5/26/2013 | 100,056 | [UNHCR report](http://data.unhcr.org/horn-of-africa/region.php?id=3&country=110) |
| Kenya | Ifo | 8/23/2015 | 7/11/2016 | 83,950 | <http://data.unhcr.org/horn-of-africa/region.php?id=3&country=110> |
| Kenya | Ifo 2 | 9/4/2011 | 1/20/2012 | 65,442 | <http://data.unhcr.org/horn-of-africa/region.php?id=3&country=110> |
| Kenya | Ifo 2 | 4/21/2013 | 5/26/2013 | 64,789 | UNHCR reports (split into Ifo 2 east and west - combined) |
| Kenya | Ifo 2 | 8/7/2015 | 4/25/2016 | 49,940 | http://data.unhcr.org/horn-of-africa/region.php?id=3&country=110 |
| Kenya | Ifo 3 | 9/11/2011 | 1/17/2012 | 37,115 | https://data.humdata.org/dataset/hoa-refugee-database |
| Kenya | Kakuma | 9/14/2009 | 11/30/2009 | 62,015 | www.jidc.org/index.php/journal/article/download/1966/694 |
| Kenya | Kambioos | 10/27/2011 | 2/10/2012 | 11,361 | <http://data.unhcr.org/horn-of-africa/region.php?id=3&country=110> |
| Kenya | Kambioos | 7/16/2015 | 4/18/2016 | 19,671 | <http://data.unhcr.org/horn-of-africa/region.php?id=3&country=110> |
| Niger | Ayorou | 7/1/2012 | 7/15/2012 | 9,189 | UNHCR outbreak report |
| Niger | Mangaize | 7/31/2012 | 9/18/2012 | 5,549 | UNHCR outbreak report |
| Niger | Mangaize | 5/13/2013 | 5/13/2013 | 8,004 | UNHCR outbreak report |
| Niger | Tabareybarey | 5/7/2013 | 5/24/2013 | 8,819 | UNHCR outbreak report |
| Republic of Congo | Betou | 1/25/2012 | 6/6/2012 | 37,333 | http://popstats.unhcr.org/en/demographics |
| Republic of Congo | Impfondo | 1/27/2012 | 6/1/2012 | 21,140 | http://popstats.unhcr.org/en/demographics |
| Republic of Congo | Liranga | 1/2/2012 | 10/1/2012 | 19,396 | http://popstats.unhcr.org/en/demographics |
| South Sudan | Gorom | 6/26/2015 | 7/1/2015 | 2,754 | http://popstats.unhcr.org/en/demographics |
| Tanzania | Nyarugusu | 9/30/2009 | 11/9/2009 | 60,971 | http://lib.ugent.be/fulltxt/RUG01/001/491/185/RUG01-001491185_2011_0001_AC.pdf |
| Tanzania | Nyarugusu | 5/14/2015 | 6/3/2015 | 155,000 | MSF sitrep (19/05/2015). |
| Tanzania | Kagunga | 5/10/2015 | 5/27/2015 | 50,000 | sitrep 5/19/2015 |
| Tanzania | Kigoma transit center | 5/19/2015 | 5/27/2015 | - |  |
| Thailand | Don Yang | 10/6/2011 | 10/6/2011 | 4,144 | http://www.theborderconso rtium.org/media/11778/2011-10-oct-map-tbbc-unhcr-1-.pdf <http://www.theborderconsortium.org/resources/key-resources/> |
| Thailand | Mae La | 5/24/2010 | 11/8/2010 | 46,078 | <http://www.theborderconsortium.org/resources/key-resources/> |
| Thailand | Nu Po | 9/20/2015 | 10/11/2016 | 10,461 | <http://www.theborderconsortium.org/resources/key-resources/> |
| Thailand | Tham Hin | 10/16/2011 | 10/16/2011 | 7,796 | <http://www.theborderconsortium.org/resources/key-resources/> |
| Thailand | Umpiem Mai | 4/26/2010 | 4/26/2010 | 17,697 | <http://www.theborderconsortium.org/resources/key-resources/> |
| Uganda | Adjumani | 8/9/2016 | 9/25/2016 | 30,000 | http://www.unhcr.org/57b5a3544.html |
| **OVERALL** |  | **9/14/2009** | **10/11/2016** | **1,559,243** |  |
